# Does physical activity prevent cognitive decline and dementia?: A systematic review and meta-analysis of longitudinal studies

**DOI:** 10.1186/1471-2458-14-510

**Published:** 2014-05-27

**Authors:** Sarah J Blondell, Rachel Hammersley-Mather, J Lennert Veerman

**Affiliations:** 1 The University of Queensland, School of Population Health, 4006 Herston, Queensland, Australia; 2 Counselling and Health at Student Services, University of Southern Queensland, 4350 Toowoomba, Queensland, Australia

**Keywords:** Physical activity, Exercise, Cognitive decline, Dementia, Cognitive impairment, Alzheimer’s disease, Cognition

## Abstract

**Background:**

By 2050, it has been estimated that approximately one-fifth of the population will be made up of older adults (aged ≥60 years). Old age often comes with cognitive decline and dementia. Physical activity may prevent cognitive decline and dementia.

**Methods:**

We reviewed and synthesised prospective studies into physical activity and cognitive decline, and physical activity and dementia, published until January 2014. Forty-seven cohorts, derived from two previous systematic reviews and an updated database search, were used in the meta-analyses. Included participants were aged ≥40 years, in good health and/or randomly selected from the community. Studies were assessed for methodological quality.

**Results:**

Twenty-one cohorts on physical activity and cognitive decline and twenty-six cohorts on physical activity and dementia were included. Meta-analysis, using the quality-effects model, suggests that participants with higher levels of physical activity, when compared to those with lower levels, are at reduced risk of cognitive decline, RR 0.65, 95% CI 0.55-0.76, and dementia, RR 0.86, 95% CI 0.76-0.97. Sensitivity analyses revealed a more conservative estimate of the impact of physical activity on cognitive decline and dementia for high quality studies, studies reporting effect sizes as ORs, greater number of adjustments (≥10), and longer follow-up time (≥10 years). When one heavily weighted study was excluded, physical activity was associated with an 18% reduction in the risk of dementia (RR 0.82; 0.73-0.91).

**Conclusions:**

Longitudinal observational studies show an association between higher levels of physical activity and a reduced risk of cognitive decline and dementia. A case can be made for a causal interpretation. Future research should use objective measures of physical activity, adjust for the full range of confounders and have adequate follow-up length. Ideally, randomised controlled trials will be conducted. Regardless of any effect on cognition, physical activity should be encouraged, as it has been shown to be beneficial on numerous levels.

## Background

### Cognitive decline

By 2050, it has been estimated that the world will number 2 billion older adults, aged ≥60 years, or approximately one-fifth (22%) of the population [1]. Such a demographic profile will have significant implications on a multitude of dimensions, including social, economic and health [1]. Individuals aged ≥65 years are over twice as likely as a 20 year old to be afflicted with ≥ 1 chronic disease, 84 per cent compared to 38 per cent respectively, and other physical and mental disturbances [2]. One particular issue with potentially significant implications for all of the aforementioned factors, is cognitive decline. Research suggests that cognitive decline is associated with the aging process, particularly from age ≥50 years [3]. Whilst some cognitive decline, stemming from reduced brain size and plasticity, is normally occurring in most people, not all cognitive decline is considered ‘normal’ [4,5]. The following will outline, in order of severity, ‘abnormal’ cognitive decline.

### Cognitive impairment no dementia (CIND)

CIND refers to the subjective and objective cognitive decline, which is neither normal nor extreme, experienced by individuals, and which is not sufficient to warrant a diagnosis of dementia [6]. This form of cognitive decline is not debilitating but, nevertheless, causes worry to the sufferer [6]. The criteria for CIND diagnoses are not yet universally agreed upon, but generally relate to a decline in cognitive domains, such as memory, executive function or praxis, that is deemed worse than that of individuals in the same age bracket, of the same sex and educational level [7]. A CIND diagnosis may be made regardless of known underlying mechanisms, such as delirium, substance abuse and psychiatric illness [8]. Approximately one-fifth of Americans aged ≥ 71 years are thought to suffer from CIND [9]. CIND may be a stand-alone illness or may be a precursor to dementia [9]. A significant proportion, namely half to two-thirds, of those individuals fitting the CIND classification may be further classified as having MCI [10].

### Mild cognitive impairment (MCI)

MCI is an ever- evolving term; however, it is most widely used to categorise older adults who present with subjective and objective memory impairment that cannot be explained by an underlying neurological or psychiatric ailment, and whose other cognitive functions are generally intact with no impediment to their everyday activities and life [10]. MCI is considered a precursor to dementia and AD.

Neuroimaging of people with MCI has shown early structural changes that are in keeping with what would be expected of AD sufferers [10]. Annually, 10 to 15 per cent of individuals with MCI progress to dementia, as compared to 1 to 2 per cent of healthy adults, and this figure is thought to be on the rise [6]. It is important to note from this that clinically-recognised abnormal cognitive decline represents only an increased risk for dementia, and does not mean that dementia is inevitable; in fact, people may revert back to a previous unimpaired state [11]. The identification of cognitive impairment before full-blown/clinically diagnosed dementia may, however, provide the opportunity for possible preventative measures.

### Dementia

Dementia, like CIND and MCI, is an illness primarily seen and diagnosed in older adulthood; some 90–98 per cent of cases are aged ≥65 years [1]. Dementia is a syndrome that is typified by memory impairment, as well as ≥1 other cognitive deficit [12]. Dementia may be diagnosed if these deficits (1) are incapacitating for the individual, i.e. he/she is no longer able to function in daily tasks; (2) represent a decline from previous functioning; and (3) are not attributable exclusively to a delirium, i.e. a short-onset disturbance in cognition [12]. A number of categories of dementia exist and represent a number of different underlying factors, including dementia with Lewy bodies, vascular dementia(VaD) and Alzheimer’s disease (AD), with the latter two sub-types being the most common [13,14].

In 2010, the number of people with dementia was reportedly 35.6 million, with AD and VaD accounting for 80 per cent of all clinically diagnosed dementia cases [1,14]. There are currently no curative treatment options for sufferers of dementia [15]. Given that, at its extremes, dementia may cause a sufferer to forget key biographical information, including his/her own name and family members, it is not surprising that people with dementia often require some significant degree of care [12]. In 2010, it was estimated that the world-wide economic cost of dementia was US$604 billion, the vast majority of which was not provided by the formal sector, but rather by unpaid informal carers, i.e. spouse, child [1,5]. Dementia, therefore, is a huge burden for sufferers, their carers, families, friends and the wider community.

Given the high prevalence of dementia, limited treatment options, the costs of the disease to individuals, families and society, and the increased risk of a subset of those with CIND to develop dementia, it is important to explore potential risk and protective factors for both cognitive decline and dementia.

### Risk and protective factors for cognitive decline and dementia

Given the apparent connectedness between cognitive decline and dementia, it seems fitting that they would share many of the same risk and protective factors. Both cognitive decline and dementia share common risk factors. Some of these are amenable to change, others not. Non-modifiable factors associated with both cognitive decline and dementia include age, sex and genetics (particularly APOE genes) [11,16]. Whilst it is important to be aware of these, from a preventive point of view, the focus needs to be on measures that are modifiable. Cardiovascular risk factors, including hypertension, diabetes, the metabolic syndrome, obesity and smoking are considered as risk factors for both cognitive decline and dementia [1,11,15]. There is suggestive evidence that lifestyle factors may contribute to, or be protective of, cognitive decline and dementia, one such example being physical activity [7].

### Physical activity as a protective factor for cognitive decline and dementia

Physical activity is defined as the movement of skeletal muscles, resulting in energy expenditure exceeding the resting state [17]. Physical activity, which encompasses exercise, is different from physical fitness [18]. One can be physically active without necessarily having high aerobic fitness [19]. Physical activity is noted as being beneficial across many domains, including cardiovascular disease, cancer and depression [5].

Much research has been undertaken to assess whether physical activity reduces cognitive decline and prevents dementia. Results from prospective studies have shown mixed results [20,21]. Aarsland et al. 2010, for example, noted that of five studies looking into physical activity and cognitive decline, two reported a significant relationship for both sexes, two reported a significant relationship for females only, and one showed no association [16]. A similar picture emerges for dementia - Aarsland et al. 2010 found that, of 12 studies examining physical activity and dementia, over half did not find statistically significant effects [16]. However, despite individual variation between studies, systematic reviews and meta-analyses of longitudinal studies do lend support to the association between physical activity, cognitive decline and dementia.

Sofi et al. 2011 [20] undertook a systematic review and meta-analysis of prospective studies exploring physical activity and risk of cognitive decline in non-demented individuals. Cognitive decline, or cognitive impairment, was defined as decline in tests of cognitive function at follow-up (no minimum threshold was specified). Participants with high and low-to-moderate levels of physical activity were compared to those who were sedentary. Included as part of the meta-analyses were 12 prospective studies (15 cohorts) published up until January 2010 [22-33]. All studies, bar one, had participants aged ≥65 years. Both low-to-moderate level (HR 0.65, 95% CI 0.57- 0.75; p = <0.001) and high level (HR 0.62, 95% CI 0.54-0.70; p = <0.001) physical activity were shown to be protective of cognitive decline.

Hamer & Chida 2009 [34] explored the relationship between physical activity and neurodegenerative diseases in healthy adults, at baseline, in meta-analyses of prospective cohort studies. The outcome measure was a diagnosis of dementia or cognitive impairment at follow-up. Included as part of the meta-analyses were nine studies on dementia and AD (10 cohorts), published up until 2007 [23,35-42]. The highest level of physical activity, when compared with the lowest level, gave a relative risk (RR) of 0.72 (95% CI 0.60 - 0.86, p = <0.001) for dementia and 0.55 (95% CI 0.36 - 0.84, p = <0.006) for AD.

The following review will summarise results from prospective studies into physical activity and cognitive decline, and physical activity and dementia up to January 2014. The present review is undertaken in view of additional original research in the field, including a number of studies that include objective measures of physical activity. This review also utilises a quality assessment tool to improve the validity of results [43]. Examined in this review are individuals aged ≥40 years who were in good health and/or randomly selected from the community.

## Methods

This paper uses the preferred reporting items for a systematic review and meta-analysis (PRISMA) to ensure accuracy and comprehensiveness [44]. A review protocol was written prior to undertaking the searches. The paper has been registered with PROSPERO, registration number CRD42014008722.

### Search strategy

This review builds on the work of the two most recently published systematic reviews on physical activity and cognitive decline and dementia. Sofi et al. 2011 [20] explored the relationship between physical activity and cognitive decline and Hamer & Chida 2009 [34] looked at the association between physical activity and dementia. An updated database search retrieved papers published after these reviews. Two modes, therefore, as outlined below, were used to search for relevant literature.

### Previous systematic reviews

Previous systematic reviews on physical activity preventing cognitive decline or dementia were included in the current review. The reference lists of the Sofi et al. 2011 [20] and Hamer & Chida 2009 [34] articles were examined, and relevant articles sought, providing evidence up until 2010 and 2007, respectively.

### Update of database search

Searches were conducted to identify any relevant studies published since 2007 (the cut-off date for the earliest literature searches from the previous reviews). The PubMed and PsycInfo databases were searched for studies published between 1 January 2007 and 31 December 2013. Terms searched were ((“physical activity” OR “exercise”) and (“cognitive decline” OR “dementia” OR “cognitive impairment” OR “Alzheimer’s disease” OR “cognition”)). The search was further limited, using filters available in the databases, to journal articles with subjects aged ≥ 40 years.

### Study selection

Study selection was limited to those (1) utilising a prospective design; (2) utilising a population-based sample; (3) with a definition of what constitutes cognitive decline or dementia, and a description of the methods used to assess these; (4) with data on baseline physical activity; (5) original research articles from dates after identified/included reviews; and (6) that reported estimates of association between physical activity subgroups and cognitive decline or dementia.

### Data extraction

Key data from the relevant studies were independently extracted and tabulated by two investigators with a master-level degree in health sciences (SJB, RHM). Inclusion of studies was based firstly, on title; secondly, on abstract; or thirdly, on full text. The PRISMA 2009 flow diagram was used as a template for reporting study inclusion [44]. Discussion was undertaken post-extraction by the investigators to resolve any differences of opinion. Where agreement could not be reached, a third investigator with a PhD (JLV), was consulted. Additional file 1: Table S1 presents the extracted information.

### Quality assessment

Included studies were assessed by the two independent reviewers, as described above, for methodological quality with the use of a tool adapted from Singh et al. 2012 [45]. Additional file 2: Table S2 contains the ten items that were used to assess study quality. Each of the items was weighted equally, with a score of one representing a ‘yes’, and zero a ‘no’. Where a paper cited other papers (for further details) the additional papers were sought; however, if the secondary papers did not contain the required information, no additional papers were sought. In such instances the criterion in question was given a score of ‘0’, for not being stated. If a paper used a standardised measure, and did not report the reliability or validity, a cursory search of Google Scholar (using the measure name and ‘validity’ or ‘reliability’) was undertaken. If the required information could not be readily accessed (in the first page of search results), then a ‘0’ was given. A score (out of ten) was allocated to papers by each reviewer, and any points of difference were discussed until an agreement could be met. Where an agreement could not be met, a third investigator, as above, was consulted. In line with Singh et al. 2012, studies with a score equating to ≥70 per cent were considered as “high quality”, and those <70 per cent were deemed “low quality” studies [45].

### Statistical analysis

MS Excel with add-in **MetaXL** [freely available at http://www.epigear.com/index_files/metaxl.html] was utilised to synthesise the effect sizes from individual studies, with the most fully-adjusted included in the meta-analyses. All effect sizes were reported in their original format, and outputs given as relative risks (RRs). Analyses were carried out on the highest level of physical activity compared to the lowest level of physical activity for cognitive decline and dementia, separately, as this was how most studies reported their results. Heterogeneity was examined in multiple ways. Firstly, it was assessed via the I^2^ statistic, whereby an I^2^ statistic of 25 per cent was considered low; 50 per cent, moderate; and 75 per cent, high [46]. Secondly, sensitivity analyses were conducted to address possible sources of heterogeneity. Potential publication bias was assessed with the use of funnel plots.

## Results

### Search outputs

#### Previous reviews

**Figure 1:**
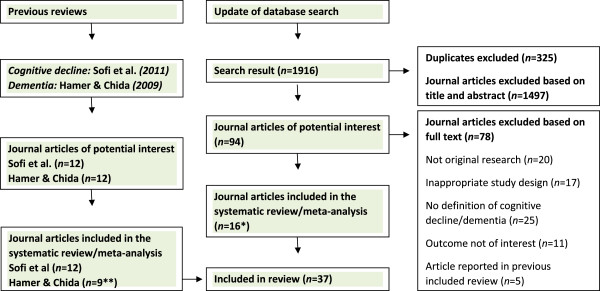
**Flow diagram of search strategy.** *Note – one paper [50] assesses both cognitive decline and dementia and is thus included in both analyses. **Three papers identified in the review for physical activity and dementia were excluded due to ‘outcome not of interest’.

Twelve prospective studies (15 cohorts) [22-33] were obtained from the Sofi et al. 2011 [20] systematic meta-analysis on physical activity and risk of cognitive decline, see Figure 1. Nine studies on dementia and AD (10 cohorts) [23,35-42] were derived from the systematic review by Hamer & Chida 2009 [34] on physical activity and neurodegenerative diseases (including dementia).

#### Database search

The searches yielded 1916 titles. Of those, 94 potentially relevant papers where identified and full text articles retrieved; see Figure 1. Twenty studies were not original research, seventeen papers had inappropriate study design, no definition of cognitive decline/dementia was given in twenty-five studies, eleven studies reported on outcomes not of interest, and five articles identified were reported in the previous reviews. Sixteen studies met the inclusion criteria and are included in the review [21,47-61]. One small study reported only ORs for the combined condition MCI/AD (*Active vs inactive* OR: 0.93; 95% CI 0.45-1.90) [62]. Inclusion of this study did not change the results in either analysis, and was excluded from the analysis/not reported (“outcome not of interest”).

### Study characteristics

#### Cognitive decline

Assessed were 17 prospective studies (21 cohorts), 12 studies from the Sofi et al. review paper [20] and 5 additional studies identified in the database search [47-51], on physical activity and cognitive decline, see Additional file 1: Table S1. The majority of the cohorts were mixed (*n* = 9); followed by male only (*n* = 7) and female only (*n* = 5). Studies were conducted world-wide, including China, Italy, Netherlands, Australia, Canada, Singapore, Germany, Japan, UK and USA. The sample sizes ranged from 27 to 12303. Follow-up time ranged from 1 to 21 years. Cognitive decline was assessed primarily with the MMSE, 8 cohorts, and the modified MMSE, 5 cohorts. Assessment of physical activity was undertaken via the use of questionnaires, with the exception of one study that used doubly-labelled water and indirect calorimetry [49]. All studies adjusted for confounders, the number of factors ranging from 1 to 18. Sixteen cohorts controlled for at least age and education, two controlled for age and National Reading Test score, two controlled for age and education only, respectively, and one controlled for neither.

#### Dementia

Twenty-one studies on physical activity and dementia (26 cohorts) were examined in relation to physical activity and dementia. Nine studies were from the previous review paper [34] and twelve additional studies were identified in the database search [21,50,52-61] (Additional file 1: Table S1). The majority of the cohorts were mixed sex (*n* = 18); however, some studies reported data for males only (*n* = 7) and females only (*n* = 1). Studies represented countries globally, including Finland, Italy, UK, Australia, Korea, Iceland, Canada, France, Nigeria, the Netherlands, Japan, Hawaii and USA. The sample sizes ranged from 469 to 4945. Follow-up time ranged from 1 to 26 years. Dementia was primarily assessed with the use of a version of the DSM. With the exception of one study that utilised data from an Actigraph [52], physical activity was assessed with the use of questionnaires. Age and education were almost universally adjusted for in all cohorts, age and education (n = 20), age and National Reading Test score (n = 2), age (n = 2), education (n = 1), and neither (n = 1). Other co-variates adjusted for across the studies were broadly classified as demographics, health indicators, gene type and lifestyle factors.

#### Quality assessment

A total of eight studies of physical activity and cognitive decline were defined as being of high quality and thirteen studies were deemed as low quality. Fifteen studies on physical activity and dementia were high quality, and the remaining eleven were low quality, see Additional file 3: Table S3.

### Meta-analysis

In light of the heterogeneous nature of the studies included in both analyses (See Additional file 1: Table S1), RRs are derived from a quality effects model. The quality effects model is used when studies differ in relation to study design [43].

#### Cognitive decline

**Figure 2:**
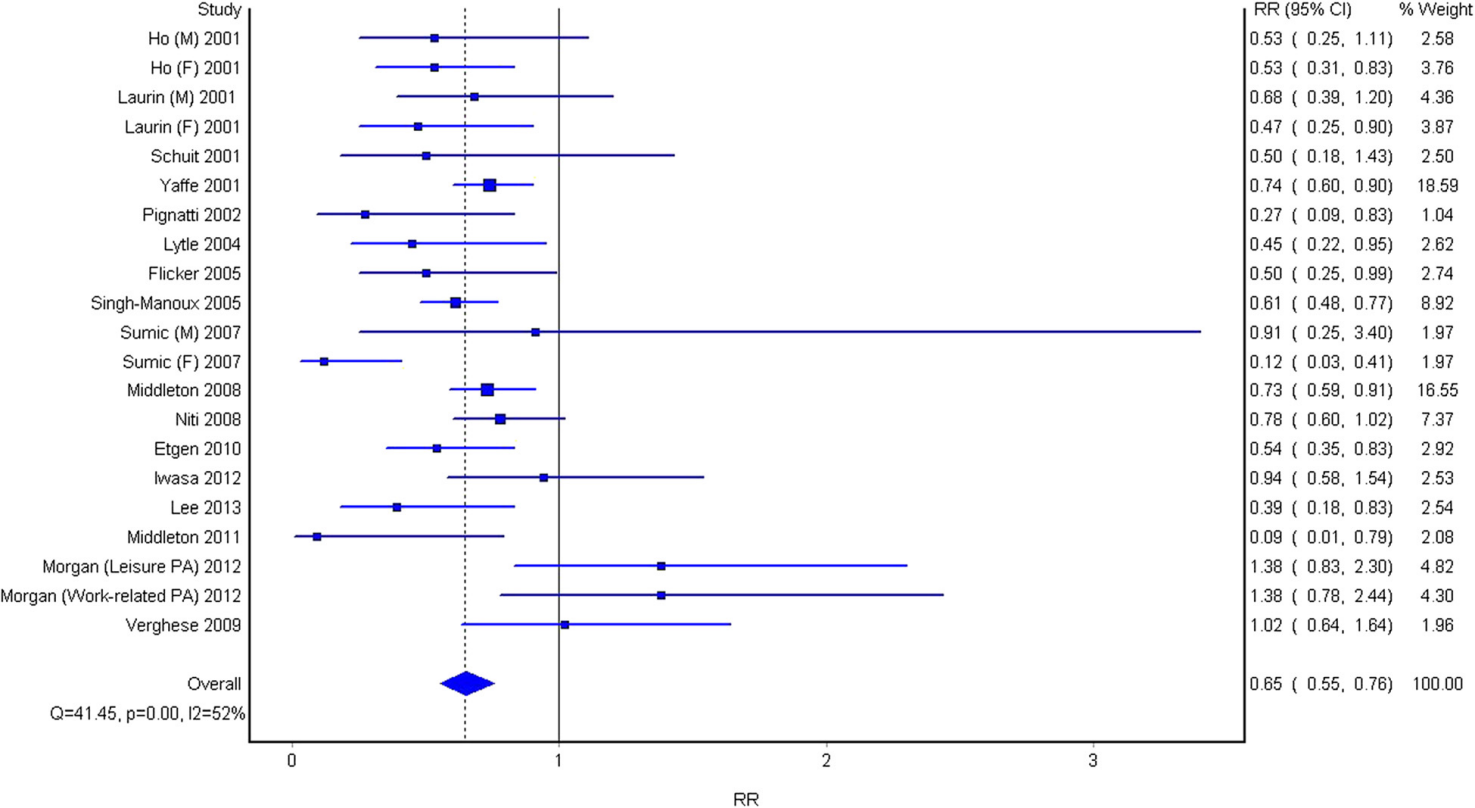
The association between high physical activity and cognitive decline.

Results suggest that participants with higher levels of physical activity, when compared to those with lower levels, are protected against cognitive decline (RR 0.65, 95% CI 0.55-0.76), as shown in Figure 2. There was moderate heterogeneity between the studies (I^2^ = 52%).

#### Dementia

**Figure 3:**
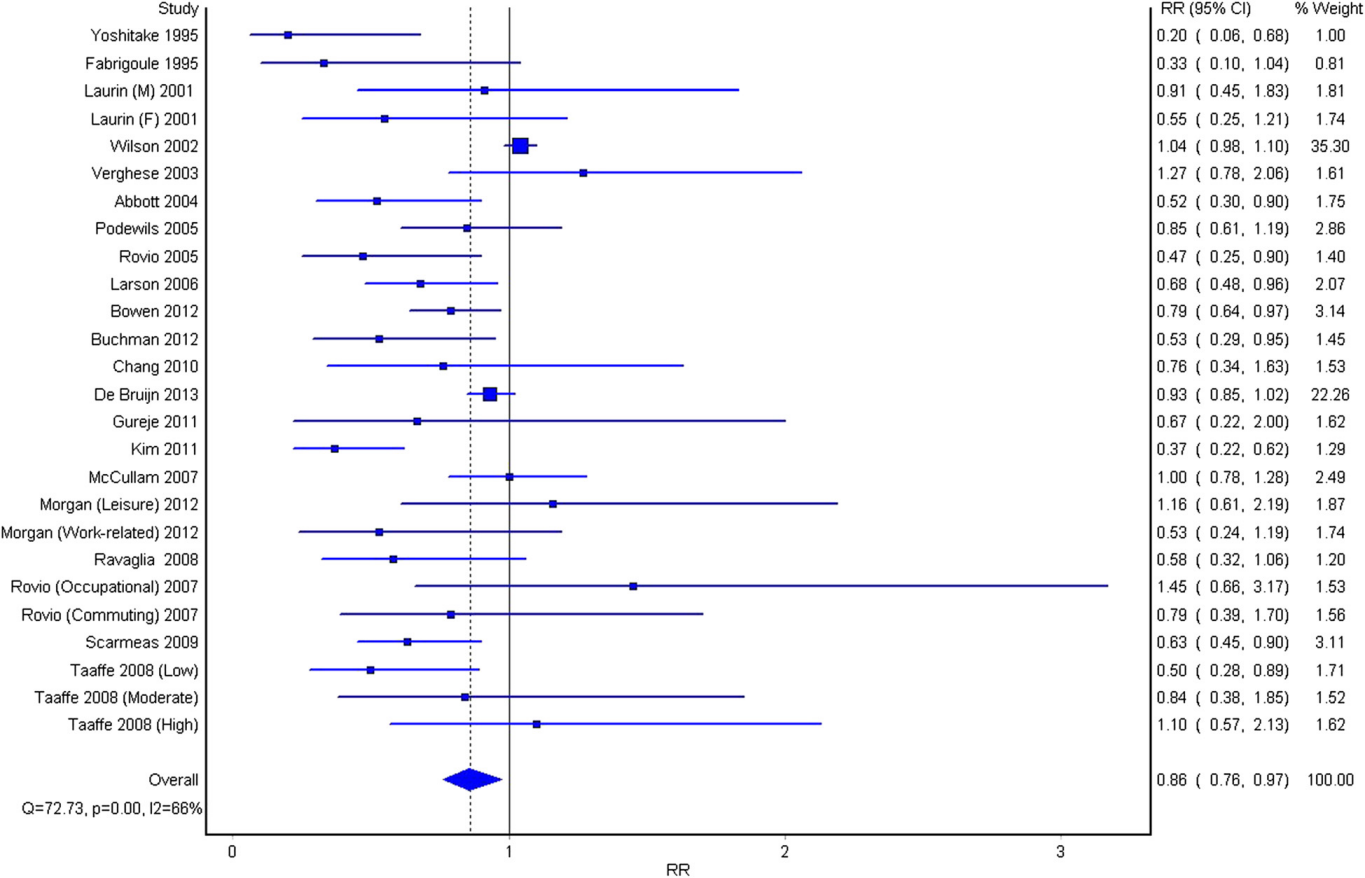
The association between high physical activity and dementia.

Results suggest that higher levels of physical activity, versus lower levels of physical activity, are associated with a 14% reduction in the risk of dementia (RR 0.86, 95% CI 0.76-0.97), see Figure 3. Heterogeneity between the studies was high (I^2^ = 66%).

### Sensitivity analyses

Sensitivity analyses were performed in view of the non-homogenous nature of studies in both domains. Studies were grouped by low quality or high quality, ORs or RRs, follow-up time <10 or ≥ 10 years, and number of adjustments <10 or ≥ 10 (see Additional file 4: Table S4). For both cognitive decline and dementia, studies reporting ORs, RR 0.67 and RR 0.89, studies of high quality, RR 0.73 and RR 0.87, with a greater number of adjustments (≥10), RR 0.68 and RR 0.86, and longer follow-up time (≥10 years), RR 0.89 and RR 0.86, respectively, provided more conservative findings. An additional sensitivity analysis was run for the dementia studies by excluding one small study [37] that was weighted heavily in the quality effects model due to its small confidence intervals. Exclusion of this study (Wilson et al. 2002 [37]) strengthened the negative association of physical activity with dementia, RR 0.82 (0.73 - 0.91).

### Publication bias

**Figure 4:**
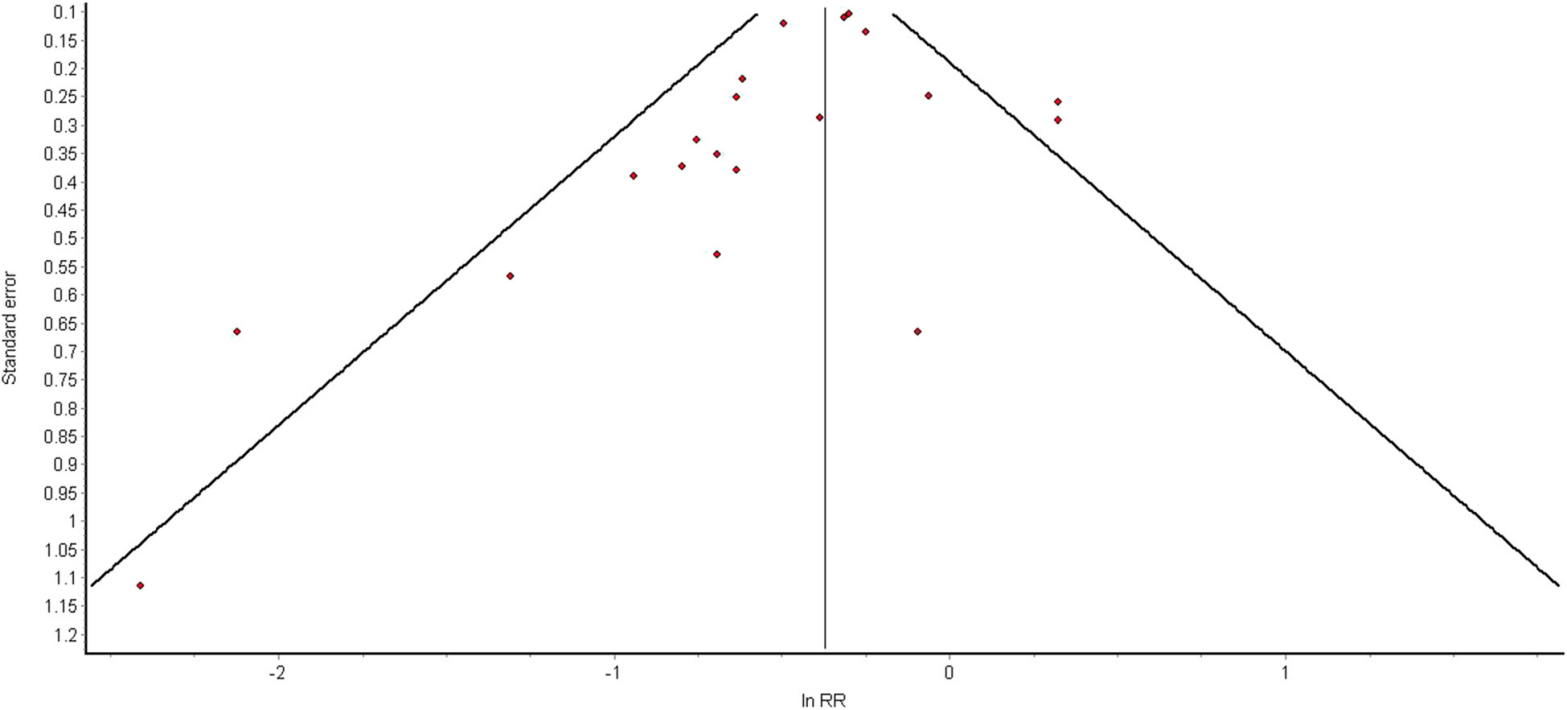
Funnel plot for studies on high physical activity and cognitive decline.

**Figure 5:**
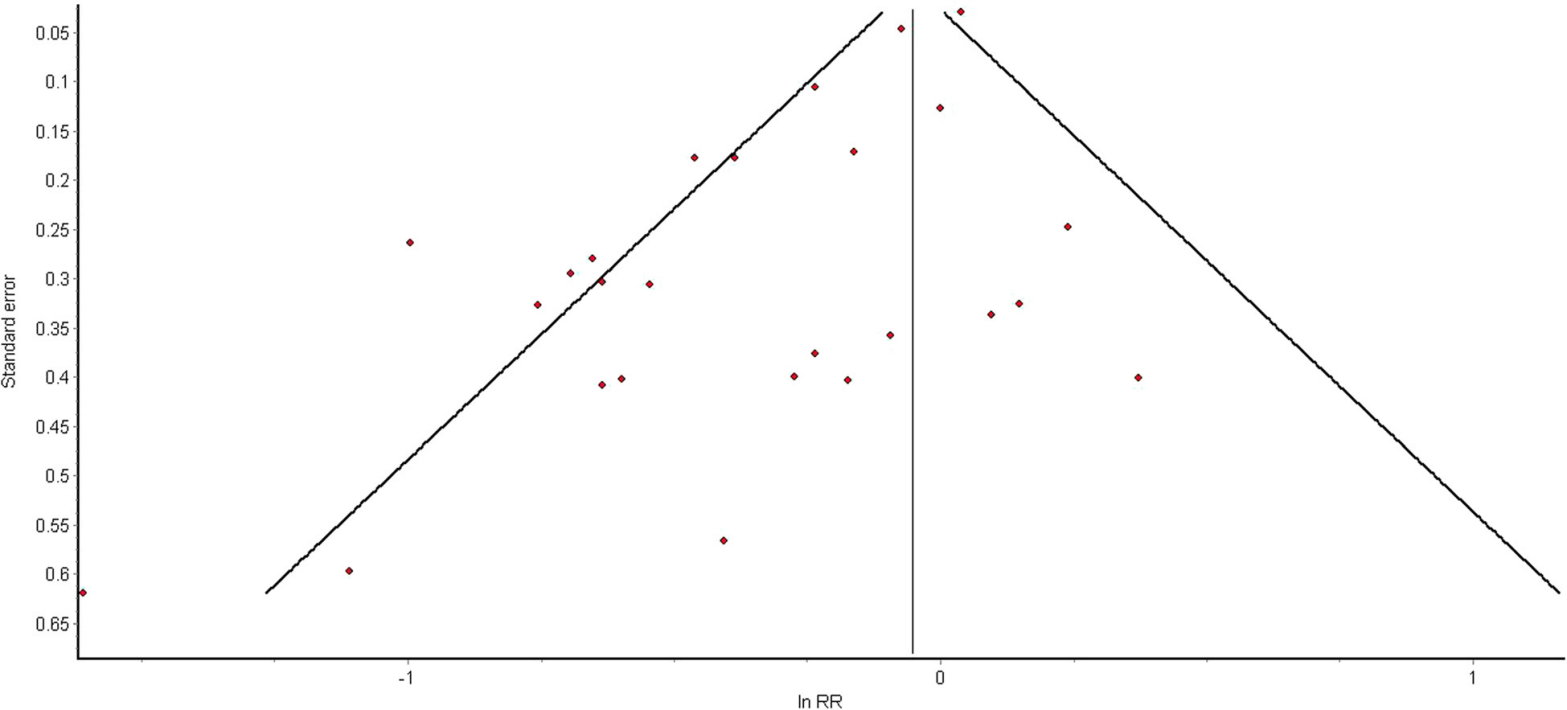
Funnel plot for studies on high physical activity and dementia.

Publication bias was assessed via funnel plots, see Figures 4 and 5. The funnel plot for cognitive decline does not suggest publication bias, but publication bias may have influenced the findings for dementia, where the heavily-weighted study by Wilson (2002) found no effect of physical activity [37].

## Discussion

The aim of this article was to review, update and quantify the association between physical activity, cognitive decline and dementia. The current review shows significant negative associations of physical activity with cognitive decline and dementia, with overall effects of RR 0.65, 95% CI 0.55-0.76 and RR 0.86, 95% CI 0.76-0.97, respectively. The addition of more recent studies to the previous reviews marginally diminished the relationship between physical activity and cognitive decline, and physical activity and dementia. Publication bias may have influenced the results for dementia. Excluding the study by Wilson et al. [37], put the effect of physical activity on dementia as stronger than those reported in the overall findings. This analysis brought results for dementia more in line with those witnessed for cognitive decline. Unlike most authors, Wilson et al. [37] analysed hours of physical activity per week and dementia, not high versus low physical activity. This resulted in (1) more efficient use of the data, and thus (2) more power, (3) a lesser effect, but smaller confidence intervals and, therefore, a weight of approximately 35 per cent in the quality-effects analysis. While this analysis clearly differs from the others, our pre-defined selection criteria, however, provided no grounds to remove the study (despite it being a small study that was weighted heavily in the analysis). On the whole the results do, however, lend support to the notion of a causal relationship between physical activity, cognitive decline and dementia, according to the established criteria for causal inference.

### Bradford Hill criteria

Austin Bradford Hill first established guidelines for causality, which is today often considered the leading method for identifying a causal relationship [63]. It is important to note that Bradford Hill himself did not present these factors as needing to be strictly adhered to, but rather as aids for interpreting associations [64]. The Bradford Hill criteria are (1) strength of association; (2) consistency; (3) specificity; (4) temporality; (5) biological gradient; (6) plausibility; and (7) coherence [65]. While many of these factors are not infallible, the proposed considerations are still useful [65]. In the following paragraphs, we will discuss each in turn.

### Association

This study confirms that there is an association between physical activity and cognitive decline and dementia. In terms of strength, Webb & Bain 2011 [66] suggest that RR 0.20 should be considered strong, and RR 0.50 considered moderately strong. In line with this, then, the associations established by this review may be considered as low-to-moderate, although the magnitude may in fact be inaccurate due to several factors, for example publication bias.

Publication bias refers to the under-reporting of results that fail to find significant positive associations [46]. The funnel plot for cognitive decline does not suggest publication bias. However, publication bias may have influenced the findings for dementia, whereby a large number of smaller studies show a larger-than-average effect. Misleading effect sizes may stem also from inadequate control for confounders.

A number of variables have been identified as possible confounders in the relationship between physical activity, cognitive decline and dementia. Scarmeas et al. 2009 [60] identify and control for a comprehensive list of variables in their study; however, a number of other studies included in the current review failed to adequately adjust their data for known confounders. Potential confounders to be controlled for include age, sex, educational level, alcohol consumption, smoking status, depression score, stress, anxiety and cardiovascular disease [4,16]. Incomplete adjustment for confounders may inflate the association between physical activity, cognitive decline and dementia and, therefore, control for confounders should be a priority for researchers. One dementia study [42] included in the meta-analysis did not adjust for age or education, both key/plausible confounders; however, exclusion of this study did not alter results significantly (RR: 0.86, 0.76-0.98). Sensitivity analysis into number of adjustments, for example, revealed that those studies which adjusted for a greater number of confounders (≥10), as compared to a lesser number (<10), found a smaller protective effect of physical activity on cognitive decline, RR 0.68 (0.51 – 0.91), and dementia, RR 0.86 (0.77 – 0.96). Inadequate control for confounders may also have implications for consistency.

### Consistency

Consistency relates to the reliability of outcomes across different settings, populations, study designs, etc. [64]. Consistency, therefore, inherently relies on sound methodological underpinnings. In the present study, inconsistent protective effects may be traced to the many methodological issues in both domains, as well as to limits in the power of individual studies and, perhaps, real differences in effects across age and other characteristics of the population under study.

Plassman et al. 2010 [7], in their systematic review of observational studies on factors associated with risk for, and possible prevention of, cognitive decline in later life, found cognitive decline to be classified in numerous ways, for example categorical or continuous. However, one test is used predominantly in the literature, the MMSE; in the current review, eight studies utilised the MMSE to assess cognitive decline. The MMSE, a brief 30-item cognitive test, has been the most widely used test to determine an individual’s cognitive health, in terms of possible cognitive impairment or dementia. Scores on the MMSE of <24 points are considered to be indicative of dementia, and >3 point score declines are in line with mild impairment [20]. However, the use of this test is increasingly being challenged, as it is considered to have insufficient scope, and, therefore sensitivity, to identify mild forms of cognitive decline [67]. A modified version of the MMSE, the 3MS, which has an altered scoring system and additional questions, was the second most widely used cognition test across the studies in the review [68]. The 3MS is noted as having slightly greater validity and reliability than the MMSE [69]. The screening/assessment of cognitive decline using tests designed to screen for dementia is, however, noted as potentially biasing persons with cognitive decline toward sub-clinical dementia [8]. The utilisation of psychometric tests not specifically designed for assessment of dementia may be more appropriate, and may limit the aforementioned bias.

So, too, it has been noted that physical activity is a somewhat ill-defined term [7]. Physical activity can be described in terms of leisure activity only, all activity, “sweat index”, etc. [4,62]. These physical activity indicators are then most often assessed via self-reported questionnaire; however, such a subjective method may introduce bias, for example recall and social desirability bias [4]. The physical activity questionnaires utilised in the papers included in the review often failed to provide sufficient detail on activity type, frequency, duration, intensity, and, therefore, limited insights can be drawn of clinical significance. Further, the majority of studies did not report information on the validity or reliability of the measures. Even when physical activity questionnaires are more in depth, however, Middleton et al. 2011 [49] note that older adults may not adequately assess their range of activities, particularly low-intensity physical activity. Inaccuracies in measurement of physical activity, as outlined above, may lead to regression dilution bias, whereby imprecise measurement of the exposure variable may bias a result toward null [70]. There is, therefore, a great need to use objective measures such as accelerometry [4]. Only two studies in the present review utilised an objective measure of physical activity. Buchman [52] used actigraphy in 716 older adults, with a follow-up time of 3.5 years, and found total daily physical activity to be significantly protective for AD (HR 0.53, 95% CI 0.29-0.95). Likewise, Middleton et al. 2011 [49], in their study of 197 subjects over a follow-up time of 5 to 8 years, found a highly protective effect of physical activity on cognitive decline, as measured via doubly-labelled water and indirect calorimetry (*high vs low* OR: 0.09, 95% CI 0.01-0.79).

### Specificity

Specificity, the notion that one agent causes one disease, is not widely apparent in the physical activity, cognitive decline and dementia domains [65]. As outlined above, numerous factors may contribute to the outcomes of interest. However, the lessened risk for cognitive decline experienced by physically active individuals with the APOE 4 gene, offers strength to the notion of causality, in regard to specificity of susceptibility [66,71]. It must be noted, however, that specificity is not central to establishing a causal relationship, unlike temporality [66].

### Temporality

Reverse causality needs to be considered in the association between physical activity, cognitive decline and dementia. Could cognitive decline and dementia lead to a lack of physical activity, rather than the reverse? It is noted, for example, that individuals with pre-clinical AD may be less inclined to undertake physical activity [1]. To overcome such potential issues around causality, longer follow-up time may be beneficial. The current review had studies with follow-up times ranging from 1 to 21 years for cognitive decline, and 1 to 26 years for dementia. Sensitivity analyses for longer (≥10 year) and shorter (<10 year) follow-up time showed that studies with longer follow-up time found weaker protective effect of physical activity for cognitive decline, RR 0.89 (0.62 – 1.27), and dementia, RR 0.86 (0.68 – 1.11). The recent study by Morgan et al. 2012 [50], with a follow-up time of 16 years, did not lend support to the protective effect of physical activity on cognitive decline or dementia. However, Chang et al. 2010 [53], with the longest follow-up time of all studies, 26 years, found a significant association of physical activity on cognitive decline. Likewise, for dementia, Rovio et al. 2005 [41] found physical activity to be protective over an extended follow-up period (*active vs sedentary* OR: 0.47, 95% CI 0.25-0.90). The inconsistencies witnessed in establishing temporality are also mirrored in relation to biological gradient.

### Biological gradient

Dose–response relationships have not been widely reported in the literature. Laurin et al., 2001 [23], for example, witnessed a dose–response relationship in relation to physical activity and cognitive decline in females, but found no dose–response relationship for physical activity and cognitive decline in males, or for either sex in relation to physical activity and dementia. In their meta-analyses, Sofi et al. 2011 [20] found no evidence of a dose–response relationship between physical activity and cognitive decline. Likewise, Hamer & Chida 2009 [34] did not find a linear-dose response relationship across studies, although such results are not unexpected. A curvilinear relationship, with diminishing returns, seems the most likely association [72]; however, more research in this area is needed. Despite the insufficient evidence for a dose–response relationship, however, there are mechanisms by which the effects of physical activity on cognitive decline and dementia are biologically plausible.

### Plausibility

A number of hypotheses have been proposed to account for the likelihood of physical activity impacting on cognitive decline and dementia. One relates to enlargement of the cognitive reserve, which results from increased brain perfusion stimulated by physical activity; another purports vascular origins, whereby cognitive decline and dementia risk are reduced due to the protective effects of exercise on cardiovascular disease, a risk factor for dementia and, more specifically, atherosclerosis; and another suggests exercise limits stress, which in turn diminishes risk for dementia [73]. These hypotheses are not only plausible, but have often proven accurate in studies [73].

### Coherence

Results from a myriad of sources, namely animal, brain plasticity, brain-imaging, epidemiological, experimental and neuropathological studies, suggest that the above hypotheses, namely cognitive reserve, vascular origins, and stress, have merit [74]. Studies utilising neuroimaging techniques have, for example, found physically active older persons to have greater brain volume than less active older individuals (2–2.5 per cent increase, per physical activity quintile) [74]. In a study of older rats, van Praag et al. 2005 [75] found that those that exercised on a running wheel showed a reversal in declines of neurogenesis (decline in neurogenesis has been implicated in cognitive decline). The proposed causal relationship between physical activity, cognitive decline and dementia is, therefore, in keeping with what is known from research into cognitive functioning more generally. The similarities observed in the RRs for cognitive decline and dementia, in the current review, offer credence to shared underlying mechanisms (which is not surprising, given that the definition of dementia centres on cognitive decline).

### Strengths

Unlike many other studies in the field, the search strategy for the current review was not limited by language barriers, which may have given a more complete picture of the available literature. The search was also independently undertaken by two investigators to ensure thorough and accurate reporting. Evidence included in the review is up to date, and includes the first studies utilising objective measures in this field. Extensive sensitivity analyses were undertaken to adequately address the heterogeneous nature of the studies.

### Limitations

A number of limitations of this study are worth noting. The quality assessment tool, and scoring method, may be considered somewhat arbitrary. It is important to note that the same score could be attained by two very different studies each missing different things. However, there was no basis to differentially weigh study quality aspects. Further limitations are in the available evidence base. The studies used a non-standardised set of methods, which makes it difficult to accurately synthesise the findings. The tendency of authors to compare between groups with high and low (or no) physical activity limits the utility of results. This method, of utilising only data from either end of the spectrum, is a less efficient use of the data compared to analysing hours of physical activity per week, for example. Effect sizes were reported in numerous formats; all outputs, however, were given in relative risks (RRs). This is not ideal; however, there was no means of accurately converting adjusted odds ratios (ORs) to RRs, for example. This approach, of taking ORs to equal RRs, and RRs to equal hazard ratios (HRs), is widely used in the literature, e.g. [16,34].

## Conclusions

The current study provides support for the association between physical activity and cognitive decline and dementia. Although methodological limitations of the current evidence base preclude the drawing of definitive conclusions, a case can be made for a causal interpretation of this association. The implications are statistically and clinically significant. It has been estimated, for example, that 3 million AD cases could be averted globally, with a 10–25 per cent shift in modifiable risk factors, including physical activity [76]. Future research should use objective measures of physical activity, adjust for the full range of known, or likely, confounders, and have adequate follow-up length. Ideally, randomised-control trials would be conducted; however, implementing constant stimulation of physical activity over a long duration may prove difficult. In the meantime, given that physical activity has been shown to be beneficial across multiple health domains, genders and ages, physical activity should be encouraged regardless of its relationship with cognitive decline and dementia [73].

## Abbreviations

AD: Alzheimer’s Disease; CI: Confidence Interval; CIND: Cognitive Impairment No Dementia; DSM: Diagnostic and Statistical Manual of Mental Disorders; HR: Hazard Ratio; ICD: International Classification of Disease; MCI: Mild Cognitive Impairment; MMSE: Mini-Mental State Exam; OR: Odds Ratio; RCT: Randomised Control Trial; RR: Relative Risk; SD: Standard Deviation; VaD: Vascular Dementia.

## Competing interests

The authors declare that they have no competing interests.

## Authors’ contributions

SJB participated in the design of the study, extracted data, performed the statistical analysis, drafted the manuscript, and approved the final manuscript. RHM participated in the data extraction and read and approved the final manuscript. JLV conceived the study, and participated in its design and coordination, and read and approved the final manuscript.

## Authors’ information

SJB has a BBeSc, GDipIH, and MPH. RHM has a BIntSt, GradDipIntlDev, MA (Research) and MHID (Health and International Development). JLV has a MD, MPH and PhD.

## Pre-publication history

The pre-publication history for this paper can be accessed here:

http://www.biomedcentral.com/1471-2458/14/510/prepub

## Supplementary Material

Additional file 1: Table S1 Study characteristics. Click here for file

Additional file 2: Table S2 Quality assessment tool. Click here for file

Additional file 3: Table S3 Study methodological quality. Click here for file

Additional file 4: Table S4 Sensitivity analyses. Click here for file
