# Supplementary material for: Does physical activity prevent cognitive decline and dementia?: A systematic review and meta-analysis of longitudinal studies
Source: BMC Public Health. 2014 May 27;14:510. doi: 10.1186/1471-2458-14-510 (PMC4064273; doi:10.1186/1471-2458-14-510)
Supplement: Additional file 1: Table S1 — Study characteristics. [file 1471-2458-14-510-S1.docx]

| Source | Country  (baseline year) | Subjects (n)ᵅ | Sex | F/up  (yrs) | Age  (yrs) | Outcome | Assessment of Cognitive performance | Assessment of Physical Activity | Physical Activity Categories | Effect Size  (95% Confidence Interval ) | Adjustments |
| --- | --- | --- | --- | --- | --- | --- | --- | --- | --- | --- | --- |
| Ho et al., 2001 [[22](#_ENREF_22)] | China  (1991-1992) | 519 | M | 3 | ≥70 | CI ( 7 cut-off point on CAPE) | Information ⁄  Orientation part of the  CAPE | Questionnaire – practice of exercise | *No Yes* | *No* vs *Yes*  OR: 1.9 (0.9 – 4.0) | Age; education |
| Ho et al., 2001 [[22](#_ENREF_22)] | China  (1991-1992) | 469 | F | 3 | ≥70 | CI : (7 cut-off point on CAPE) | Information  Orientation part of the  CAPE | Questionnaire – practice of exercise | *No Yes* | *No* vs *Yes*  OR: 1.9 (1.2- 3.2) | Age; education |
| Laurin et al., 2001 [[23](#_ENREF_23)] | Canada  (1991-  1992) | 1831 | M | 5 | ≥65 | CIND | 3MS and clinical evaluation | Questionnaire – frequency and intensity of PA | *None*  *Low*: activity not sufficient to meet Moderate or High.  *Moderate:* frequency > 3 times p.w., equal intensity to walking  *High:* frequency >3 times p.w., > intensity than walking | *None* OR: 1  *Low* OR: 0.65 (0.30 -1.38);  *Moderate:* OR:0.84 (0.53-1.34);  *High* OR: 0.68 (0.39-1.20) | Age; education level; smoking; alcohol use; use of NSAIDs;  functional ability in  basic and instrumental ADL; self-rated health;  number of chronic  health conditions |
| Laurin et al., 2001 [[23](#_ENREF_23)] | Canada  (1991-1992) | 2784 | F | 5 | ≥65 | CIND | 3MS and clinical evaluation | Questionnaire – frequency and intensity of PA | *None*  *Low*: activity not sufficient to meet Moderate or High.  *Moderate:* frequency > 3 times p.w., equal intensity to walking.  *High:* frequency >3 times p.w., > intensity than walking | *None* OR: 1  *Low* OR: 0.69 (0.41-1.16); *Moderate* OR: 0.55 (0.36-0.82);  *High* OR: 0.47 (0.25-0.90) | Age; education level; smoking; alcohol use; use of NSAIDs;  functional ability in  basic and instrumental ADL; self-rated-health;  number of chronic  health conditions |
| Schuit et al., 2001 [[24](#_ENREF_24)] | Netherlands  (1990) | 347 | M | 3 | Mean: 74.6 | CD (>3 point decline on MMSE) | MMSE | Self-administered questionnaire – frequency and duration of PA (walking, cycling, hobbies, gardening, odd jobs and sport) converted to total weekly PA in minutes | ≤30 min/d  31-60 min/d  >60 min/d | *≤30 min/d* OR: 2 (0.70-5.60)  *31-60 min/d* OR: 1.8 (0.60-5.10)  *>60 min/d OR:* 1 | Age; education; smoking; alcohol consumption; impaired cognitive functioning at baseline; disabilities ADL; self-reported health; medical history  of myocardial infarction; angina  pectoris; temporary ischemic attack; non-insulin dependent  diabetes mellitus and cerebro-vascular disease |
| Yaffe et al., 2001 [[25](#_ENREF_25)] | USA  (1986-1988) | 5925 | F | 6 to 8 | ≥65 | CD (≥3 point decline on MMSE) | Modified  MMSE (out of 26 points; higher score indicates better functioning) | Questionnaire – frequency and duration of PA converted into k/cal per week | Lowest : 0-615 k/cal p.w.  Second: 616-1323 k/cal p.w.  Third: 1324-2414 k/cal p.w.  Highest: 2415-17531 k/cal p.w. | *Lowest* OR*:* 1  *Second* OR*:* 0.90 (0.74-1.09)  *Third* OR*:* 0.78 (0.64-0.96)  *Highest* OR*:* 0.74 (0.60-0.90) | Baseline age;  education level;  health status; functional limitation; depression  score; stroke; diabetes; hypertension;  myocardial infarction;  estrogen use; smoking |
| Pignatti et al., 2002 [[26](#_ENREF_26)] | Italy | 282 | F | 12 | 70 - 75 | CD (≥1 point decline on MSQ) | MSQ | Questionnaire – walking distance p.d. | *Low*  *High:* Walking >2 km p.d. | *Low* RR: 3.7 (1.2-11.1) | Baseline MSQ |
| Lytle et al., 2004 [[27](#_ENREF_27)] | USA  (1987-1989) | 1146 | M/F | Every 2y | ≥65 | Cognitive decline (≥3 point decline on MMSE) | MMSE | Questionnaire – type, frequency and duration of PA | *No exercise*  *Low:* All other exercise not sufficient to meet “High exercise”  *High*: Aerobic exercise of ≥30 min ≥5 times p.w. | *No exercise* OR: 1  *Low* OR: 0.63 (0.39-0.997)  *High* OR: 0.45 (0.22-0.95) | Age; sex; education;  Wave 3 MMSE score;  self-rated health |
| cker et al., 2005 [[28](#_ENREF_28)] | Australia  (1996-1998) | 12,203 | M | Mean: 4.8 | ≥65 | CI (<24 score on MMSE) | MMSE | Self-reported frequency and intensity of PA | Non-vigorous  Vigorous | *Non-vigorous* HR: 1  *Vigorous* HR:  0.50 (0.25-0.99) | Age; education; diabetes;  consumption of full- cream milk; level of alcohol consumption |
| Singh-Manoux et al., 2005 [[29](#_ENREF_29)] | UK  (1985-1988) | 10,308 | M/F | Median:11 | 35-55 | CF (cognitive functioning in the lowest quintile) | Cognitive test battery (20 word free recall; Alice Heim 4-I; Phonemic fluency measure; and Semantic fluency measure. | Questionnaire – frequency, duration and intensity of PA converted to hours per week by intensity | *Low:* <2 h.p.w of moderate PA and <1 h.p.w of vigorous PA  *Moderate:* PA >Low but <High  *High:* >2.5 h.p.w of moderate PA or >1 h.p.w of vigorous activity | Alice Heim 4-I  *High* OR: 1  Moderate OR: 1.23 (0.98-1.54)  *Low* OR: 1.65 (1.30- 2.10) | Age; gender; education level; employment grade; self-rated  health; blood pressure level; cholesterol level; smoking status;  mental health status; social  network index score, and Mill Hill  Vocabulary Scale Score |
| Sumic et al., 2007 [[30](#_ENREF_30)] | USA  (1989) | 27 | M | Mean: 4.7 | ≥85 | CI (<24 score on MMSE or 0.5 on CDR) | MMSE and CDR | Questionnaire – frequency, duration and intensity of PA | *Low*: ≤4hrs p.w  *High*:> 4 hrs p.w | *Low* HR: 1  *High* HR: 0.91 (0.25-3.40) | Age; education;  Apolipoprotein allele 4 Status; cognitive  function (delayed  recall test) |
| Sumic et al., 2007 [[30](#_ENREF_30)] | USA  (1989) | 39 | F | Mean: 4.7 | ≥85 | CI (<24 score on MMSE or 0.5 on CDR) | MMSE and CDR | Questionnaire – frequency, duration and intensity of PA | *Low*: ≤4hrs p.w  *High*:> 4 hrs p.w | *Low* HR: 1  *High* HR: 0.12 (0.03-0.41) | Age; education;  Apolipoprotein allele 4 Status; cognitive  function (delayed  recall test) |
| Middleton et al., 2008 [[31](#_ENREF_31)] | Canada  (1991) | 4683 | M/F | 5 | ≥65 | CIND, VCIND, MCI | 3MS and clinical evaluation | Questionnaire – intensity and frequency of PA | *Low:* All other exercise that does not meet Mod/High  *Moderate/High:* >3 times p.w. at least the intensity of walking | *CIND:*  *Low* OR: 1  *Mod/High* OR: 0.73 (0.59-0.91) | Age; sex; education;  use of NSAIDs;  vascular risk factor index |
| Niti et al., 2008 [[32](#_ENREF_32)] | Singapore  (2004-2005) | 1635 | M/F | 1-2 | ≥55 | CD (≥1 point decline on MMSE) | MMSE | Questionnaire – usual participation in PA (number and frequency) | *No*  *Yes* | A least 1 PA  *No* OR: 1  *Yes* OR: 0.78 (0.60-1.02) | Age; gender; education;  number of medical illness; hypertension; diabetes; cardiac  diseases; stroke; smoking; alcohol drinking; physical  functional status; depression;  APOE-ε4 status; baseline MMSE |
| Etgen et al., 2010 [[33](#_ENREF_33)] | Germany  (2001-2003) | 3903 | M/F | 2 | >55 | CI (scores >7 on 6-CIT) | 6 Item Cognitive Impairment Test (6-CIT or Short Blessed Test) | Questionnaire – frequency and intensity | *No activity:* No regular PA  *Moderate activity:* <3 times p.w.  *High activity:* ≥3 times p.w. | *No* OR:1  *Moderate* OR: 0.57 (0.37-0.87)  *High* OR: 0.54 (0.35-0.83) | Age; sex; BMI;  baseline 6CIT score;  depression; alcohol; diabetes; history of  ischemic heart disease/stroke; hyperlipidemia;  hypertension; chronic kidney disease; smoking |
| Iwasa et al., 2012 [[47](#_ENREF_47)] | Japan (2002) | 567 | M/F | 5 | ≥70 | CD (≥3 decline on MMSE) | MMSE | Questionnaire – regular PA | Yes  No | Yes OR: 1  No OR: 1.06 (0.65-1.74) | Age; gender; number of years of education; presence of chronic disease; IADL; depressive symptoms; smoking; hearing deficit and baseline MMSE score. |
| Lee et al., 2013 [[48](#_ENREF_48)] | Japan (2000) | 550 | M/F | 8 | ≥60 | CD (≥3 decline on MMSE) | MMSE | Interviewer administered questionnaire – PA in last 12 months | Quartiles | *Highest quartile of light-inten-*  *sity physical activity time* OR: 0.39 (0.18–0.83) | Age, sex, educational level,  BMI, initial MMSE score, smoking  status, self-rated health, CES-D score, education level, sleep duration, occupation, hypertension, myocardial  infarction, hyperlipidemia, diabetes mellitus, stroke,  rheumatoid arthritis,  MVPA time |
| Middleton et al., 2011 [[49](#_ENREF_49)] | USA (1998-1999) | 197 | M/F | 5 or 8 | Mean: 74.8 | CI (≥1 SD decline from baseline to follow-up on 3MS) | 3MS | Doubly labelled water and indirect calorimetry | Lowest  Middle  Highest | *Lowest* OR: 1  *Middle* OR: 0.28 (0.06-1.23  *Highest* OR: 0.09 (0.01- 0. 79) | Baseline 3MS; age; sex; race; site; years of education; fat-free mass; sleep duration; self-rated health; diabetes mellitus |
| Morgan et al., 2012 [[50](#_ENREF_50)] | UK  (1984-1988) | 1005 | M | 16 | Mean: 56 | CIND  Vascular-CIND | CAMCOG and clinical assessment;  vascular-CIND:  Hachinski Ischemia Score ≥ 3 points | Questionnaire – type, frequency and duration of work-related PA and leisure time PA | Low  Moderate  High | *Work-related CIND*: Low OR: 1  *Moderate* OR: 1.15 (0.68-1.96)  *High* OR: 1.38 (0.78-2.44)  *Leisure time CIND*:  *Low* OR: 1  *Moderate* OR: 1.09 (0.64-1.83)  *High* OR: 1.38 (0.83-2.30) | Age; social class; National Adult Reading Test score; smoking status; marital status; self-reported history of vascular disease (≥1 heart attack; angina; any IHD; stroke; claudication); alcohol consumption; BMI; common mental disorder; Speilberger’s State-Trait Anxiety Index score |
| Verghese et al., 2009 [[51](#_ENREF_51)] | USA  (1980-1983) | 401 | M/F | 21 | 75-85 | VCI (≥1 SD below mean composite score; Hachinski Score ≥4.) | Digit Symbol  Substitution, Digit span (total span), and Category  Fluency tests. Hachinski ischemic score | Questionnaire – frequency of PA converted to PAS | <8  ≥8 | <8 HR: 1  ≥8 HR: 1.022 (0.635-1.642) | Sex; education; medical illnesses; and Blessed test scores |
| Yoshitake et al., 1995 [[35](#_ENREF_35)] | Japan  (1985) | 828 | M/F | 7 | ≥65 | VaD  AD | DSM-III-R; NINCDS-AIREN; NINCDS-ADRDA | Questionnaire – leisure and work PA | Physically active: daily exercise during leisure time or moderate to severe PA at work | *Physically active*  *AD* RR: 0.20 (0.06-0.68) | Sex; age; systolic blood pressure; history of stroke at entry; alcohol, low HDS score; history of diabetes |
| Fabrigoule et al., 1995 [[36](#_ENREF_36)] | France | 2040 | M/F | 1-3 | ≥65 | D  AD | DSM-III-R; NINCDS-ADRDA; Hachinski score | Questionnaire – sport or gymnastic participation; Yes/No | Yes  No | *Yes* vs *No*  RR: 0.33 (0.10-1.04) | Age; cognitive  performance (baseline  MMSE, BVRT, and IST score) |
| Laurin et al., 2001 [[23](#_ENREF_23)] | Canada  (1991-1992) | 1831 | M | 5 | ≥65 | D  VaD  AD | DSM-IV; NINCDS-ADRDA; WHO ICD-10; NINCDS-AIREN | Questionnaire - frequency and intensity of PA | *None*  *Low*: activity not sufficient to meet Moderate or High.  *Moderate:* frequency ≥ 3 p.w., intensity equal to walking.  *High:* frequency ≥3 p.w., more intensity than walking | *None* OR: 1  *Low* OR: 0.96 (0.40-2.31)  *Moderate* OR: 0.72 (0.38-1.38)  *High* OR: 0.91 (0.45-1.83) | Age; education level; alcohol; use of NSAIDs; smoking; functional ability in basic and instrumental ADL; self-rated health; number of chronic health conditions |
| Laurin et al., 2001 [[23](#_ENREF_23)] | Canada  (1991-1992) | 2784 | F | 5 | ≥65 | D  VaD  AD | DSM-IV; NINCDS-ADRDA; WHO ICD-10; NINCDS-AIREN | Questionnaire - frequency and intensity of PA | *None*  *Low*: activity not sufficient to meet Moderate or High  *Moderate:* frequency > 3 p.w., intensity equal to walking.  *High:* frequency >3 p.w., more intensity than walking | *None* OR: 1  *Low* OR: 0.63 (0.32-1.25)  *Moderate* OR: 0.87 (0.55-1.39)  *High* OR: 0.55 (0.25-1.21) | Age; education level; alcohol; use of NSAIDs; smoking; functional ability in basic and instrumental ADL living; self-rated health; number of chronic health conditions |
| Wilson et al., 2002 [[37](#_ENREF_37)] | USA  (1993) | 842 | M/F | Mean: 4.1 | ≥65 | AD | NINCDS-ADRDA | Questionnaire – frequency and duration of PA in past two weeks converted to total weekly hours | Hours per week | OR: 1.04 (0.98-1.10) | Age; education; sex; race; possession of the APOE_4 allele |
| Verghese et al., 2003 [[38](#_ENREF_38)] | USA  (1980-1983) | 469 | M/F | Median: 5.1 | 75 - 85 | D  AD  VaD  Mixed D | DSM-III-R; NINCDS-ADRDA; California ADDTC criteria. | Interviewer administered questionnaire – frequency of 11 Physical activities (physical activity score: 0 – 77) | <9  9-16  >16 | *<9 points* HR: 1  *9-16 points* HR: 1.44 (0.91-2.28)  *>16 points* HR: 1.27 (0.78-2.06) | Age; sex; education level; the presence or absence of chronic medical illnesses; base-line score on the Blessed-Information-Memory-Concentration test |
| Abbott et al., 2004 [[39](#_ENREF_39)] | USA (1991 – 1993) | 2257 | M | Mean: 7 | 71-93 | D  VaD  AD | DSM-III-R; NINCDS-ADRDA;  California ADDTC criteria. | Questionnaire – average distance walked miles/d. | Low: <0.25 mile/d  High: >2 mile/d | *<0.25* vs *>2* *mile/d* RH: 1.93 (1.11-3.34) | Age; presence of apolipoprotein 4 alleles; baseline Cognitive Abilities Screening Instrument score; decline in physical activity since mid-adulthood; physical performance score; years of education; BMI; childhood years spent living in Japan; status as a skilled professional; diabetes; hypertension; prevalent coronary heart disease; total and high-density lipoprotein cholesterol |
| Podewils et al., 2005 [[40](#_ENREF_40)] | USA | 3375 | M/F | Mean: 5.4 | ≥65 | D  VaD  AD | NINCDS-ADRDA;  ADDTC criteria | Interviewer administered questionnaire –frequency and duration of 15 types of activities over last 2 wks. Converted to k/cal p.w. | Quartile 1: <248 k/cal p.w.; Quartile 2: 248-742 k/cal p.w.; Quartile 3 743-1657 k/cal p.w.; Quartile 4: >1657 k/cal p.w. | *Quartile 1* HR: 1  *Quartile 2* HR: 1.22 (0.93-1.60)  *Quartile 3* HR: 0.94 (0.69-1.28)  *Quartile 4* HR: 0.85 (0.61-1.19) | Age; educational level; gender; ethnicity; Apolipoprotein E genotype; baseline Modified MMSE score; magnetic resonance imaging white-matter-grade score; ADL impairment; instrumental ADL impairment; Lubben Social Network Score; social support score |
| Rovio et al., 2005 [[41](#_ENREF_41)] | Finland  (1972,  1977, 1982 or 1987) | 1449 | M/F | Mean: 21 | 65-79 | D  AD | DSM-IV; NINCDS-ADRDA | Questionnaire – frequency, duration and Intensity of leisure time PA | “Sedentary” – those who participate in leisure time PA <2 p.w.  “Active” – those who participated in leisure time PA >2 p.w. | Active vs Sedentary  *D* OR: 0.47 (0.25-0.90) | Age at re-examination; sex; education; follow-up time; locomotor disorders; *APOE* _4 genotype; midlife BMI; systolic blood pressure; cholesterol; history of myocardial infarction, stroke and diabetes mellitus; smoking status; alcohol drinking |
| Larson et al., 2006 [[42](#_ENREF_42)] | USA | 1740 | M/F | Mean:6.2 | >65 | D  AD | DSM-IV; NINCDS-ADRDA | Questionnaire – frequency and duration of PA | “exercising regularly” – participant exercised ≥3 time p.w | ≥3 times p.w. vs <3 times p.w.  HR: 0.68 (0.48-0.96) | Apolipoprotein  E _4 alleles; diabetes; hypertension; cerebrovascular disease; coronary heart disease; self-rated health; physical performance;  depression; and cognitive functioning |
| Bowen, 2012 [[21](#_ENREF_21)] | USA  (1998) | 808 | M/F | Median: 5 | ≥71 | D | Neuropsychological battery  Included: measures of orientation, verbal  and visual immediate and delayed  memory, language, attention, executive  function, reading ability, and  general intellect. | Questionnaire - frequency and intensity of PA over past 12 months | 0 = no vigorous physical activity  over the course of the study  3 = 3 years of vigorous physical activity over  the course of the study. | *Vigorous PA ≥3 per week, ≥1 yr* OR: 0.79 (0.64- 0.97) | Age; educational attainment; sex; race; ApoE; BMI; stroke; diabetes; hypertension; heart disease; smoking; alcohol consumption; TICS score |
| Buchman et al., 2012 [[52](#_ENREF_52)] | USA  (2005) | 716 | M/F | Mean: 3.5 | Mean 81.6 | AD | Clinical assessment; NINCDS-ADRDA | Actigraph, measured for up to 10 days (average sum of all daily PA counts recorded) | - | *Total daily PA* HR:0.53 (0.29-0.95) | Age; sex; education; self-reported PA; frequency of social and cognitive activities |
| Chang et al., 2010 [[53](#_ENREF_53)] | Iceland | 4945 | M/F | Mean:26 | Mean:51 | D | DSM-IV | Questionnaire – participation in PA in adult life and duration converted to total hours per week | None  ≤5 hours of PA per week  >5 hours of PA per week | None *OR: 1*  *≤5 hours of PA OR:*  0.59 (0.40-0.88)  *>5 hours of PA* OR:  0.76 (0.34-1.63) | Age; sex; education; midlife BMI; systolic blood pressure; smoking; cholesterol |
| De Bruijn et al., 2013 [[54](#_ENREF_54)] | Netherlands (1997) | 4406 | M/F | Mean: 8.8 | 61-97 | D | DSM-III-R  and NINCDS-ADRDA | Adapted  version of the Zutphen Physical Activity Questionnaire - hours p.w. spent on walking, cycling,  gardening, diverse sports, hobbies,  housekeeping activities  in past 2 weeks. | Per SD increase in physical activity | HR 0.93 (0.85 - 1.02) | Age; sex; score on MMSE; low educational  Level; smoking; APOE-e4 carrier status; hypertension; BMI; diabetes;  total cholesterol; HDL-cholesterol |
| Gureje et al., 2011 [[55](#_ENREF_55)] | Nigeria (2003) | 1225 | M/F | 39 months | ≥65 | D | Clinician Home-based  Interview to assess Function (CHIF) | International Physical Activity Questionnaire – PA across leisure-time, work, transportationand household tasks | low (physically  inactive), moderate, and high levels of PA | *Low* OR: 1.5 (0.5–4.6) | Age; sex; education |
| Kim et al., 2011 [[56](#_ENREF_56)] | Korea  (2001-2003) | 518 | M/F | 2.4 | ≥65 | D | DSM-IV; NINCDS-ADRDA criteria; NINDS-AIREN criteria | Questionnaire – leisure and work PA over past month | Not at all active  Not very active  Fairly active  Very active | *Per descending category of activity* OR 2.72 (1.61-4.60) | Age; sex; education; depression; vascular risk scores; APOE genotype |
| McCallum et al., 2007 [[57](#_ENREF_57)] | Australia | 2805 | M/F | 14 | ≥60 | D | Medical records, ICD, Geriatric assessment team with access to CT scan | Questionnaire | Daily  Rarely | *Walking (daily* vs *rarely)* HR: 1 (0.78-1.28) | Marital status; education; prior history of stroke; ADL |
| Morgan et al., 2012 [[50](#_ENREF_50)] | UK  (1984-1988) | 1005 | M | 16 | Mean: 56 | D | DSM-IV, NINDS-AIREN criteria; NINCDS-ADRDA | Questionnaire – frequency and duration of work-related PA and leisure time PA | Low  Moderate  High | Work-related: Low OR: 1  Moderate OR: 0.70 (0.36-1.39)  High OR: 0.53 (0.24-1.19)  Leisure time:  Low OR: 1  Moderate OR:0.57 (0.28-1.16)  High OR: 1.16 (0.61-2.19) | Age; social class; National Adult Reading Test score; smoking status; marital status; self-reported history of vascular disease (≥1 heart attack; angina; any IHD; stroke; claudication); alcohol consumption; BMI; common mental disorder; Speilberger’s State-Trait Anxiety Index score |
| Ravaglia et al., 2008 [[58](#_ENREF_58)] | Italy  (1999-2000) | 749 | M/F | Mean 3.9 | ≥65 | D  VaD | DSM-IV, NINCDS-ADRDA criteria, NINDS-AIREN criteria | Questionnaire – Blocks and pace walked; stair climbing; frequency and duration of PA (occupational, recreational or sporting) per week, over past year | Tertiles | Total PA:  *<4774* HR: 1  *4774-8090* HR: 0.69 (0.41-1.15)  *>8090* HR: 0.58 (0.32-1.06) | Age; gender; education; APOE genotype; comorbidity; basic ADL motor disability |
| Rovio et al., 2007 [[59](#_ENREF_59)] | Finland  (1972,  1977, 1982 or 1987) | 1449 | M/F | Mean: 20.9 | 65-79 | D | DSM-IV, NINCDS-ADRDA criteria. | Questionnaire – frequency of PA (occupational, commuting) | Sedentary  Active | *Occupational*  *Sedentary* OR: 1  *Active* OR: 1.45 (0.66-3.17)  *Commuting Sedentary* OR: 1  *Active* OR: 0.79 (0.39-1.70) | Age; sex; education; follow-up time; locomotor symptoms; main occupation during life; income; leisure time and commuting PA; APOE 4 genotype; BMI; blood pressure; cholesterol; history of myocardial infarction, stroke, and diabetes mellitus; smoking status |
| Scarmeas et al., 2009 [[60](#_ENREF_60)] | USA  (1992, 1999) | 1880 | M/F | Mean: 5.4 | Mean: 77.2 | AD | DSM-III-R; NINCDS-ADRDA criteria | Questionnaire – frequency, duration and intensity of PA over 2 week period | No: median weekly level – 0 hours  Some: median weekly level - 0.1 hrs vigorous PA; 0.8 hrs moderate PA; 1.3 hrs light PA  Much: median weekly level - 1.3 hrs vigorous PA; 2.3 hrs moderate PA; 3.8 hrs light PA | *No* HR: 1  Some HR: 0.71 (0.51-0.98)  *Much* HR: 0.63 (0.45-0.90) | Cohort; age; sex; ethnicity; education; apolipoprotein E 4 allele; caloric intake; BMI; smoking; depression; leisure activities; comorbidity index; baseline Clinical Dementia Rating score; time between first dietary and first physical activity assessment |
| Taaffe et al., 2008 [61] | Hawaii (1991-1993) | 2263 | M | Mean 6.1 | 71-93 | D  AD  VaD | DSM-III-R, NINCDS-ADRDA criteria, California Alzheimer Disease and Treatment Centers  criteria | Questionnaire – Usual 24 hr physical activity (used to create index of PA) | Low PA - ≤28.7  Moderate PA - 28.8–32.4  High PA - ≥32.5 | *Low physical function, High PA* *D* HR: 0.50 (0.28–0.89  *Moderate physical function, High PA D* HR: 0.84 (0.38–1.85)  *High physical function, High PA D* HR: 1.10 (0.57–2.13) | Age; education; apolipoprotein E e4 allele status; Cognitive Abilities Screening Instrument score; BMI; midlife systolic and diastolic blood pressure; smoking status; cholesterol; hypertension; diabetes; coronary heart disease; depression |

**Table 1** Study characteristics

ᵅNumber of participants included in analysis; 3MS: Modified Mini Mental State Examination; AD: Alzheimer’s Disease; ADDTC: Alzheimer’s disease diagnostic and treatment centers; ADL: Activities of daily living; BMI: Body Mass Index; CAPE: Clifton Assessment Procedure for the Elderly; CD: Cognitive Decline; CDR: Clinical Dementia Rating scale; CI: Cognitive Impairment; CIND: Cognitive Impairment No Dementia; D: Dementia; DSM: Diagnostic and Statistical Manual of Mental Disorders; HDS: Hasegawa's dementia scale; HR: Hazard Ratio; ICD: International Classification of Diseases; K/Cal: Kilo-calories; Mixed D: Mixed Dementia; MMSE: Mini Mental State Examination; MSQ: Mental Status Questionnaire; NINCDS-ADRDA: National Institute of Neurological and Communicative Disorders and Stroke and the Alzheimer’s Disease and Related Disorders Association; NINDS-AIREN: National Institute of Neurological and Communicative Disorders and Association Internationale pour la Recherche et l’Enseignement en Neurosciences; NSAIDs: Non-Steroidal Anti-Inflammatory Drugs; PA: Physical Activity; p.d: per day; p.w: per week; SD: Standard Deviation; VaD: Vascular Dementia; VCI: Vascular Cognitive Impairment
