# Supplementary material for: Does physical activity prevent cognitive decline and dementia?: A systematic review and meta-analysis of longitudinal studies
Source: BMC Public Health. 2014 May 27;14:510. doi: 10.1186/1471-2458-14-510 (PMC4064273; doi:10.1186/1471-2458-14-510)
Supplement: Additional file 2: Table S2 — Quality assessment tool. [file 1471-2458-14-510-S2.docx]

**Table S2** Quality assessment tool

| Quality criteria | Quality score |
| --- | --- |
| Is the participation rate ≥ 70% | ≥70% = 1 |
|  | <70% or not stated = 0 |
| Is the follow up duration ≥5 years | ≥5 years = 1 |
|  | < 5 years = 0 |
| Is the follow up rate 70% or more of the participants at baseline | ≥70% = 1 |
|  | <70% or not stated = 0 |
| Are drop outs comparable with follow-up participants | Drop outs are comparable = 1 |
|  | Drop outs not comparable = 0 |
| Was the validity of the measure assessing physical activity reported | Validity of physical activity measure reported (or can be reasonably acquired externally) = 1 |
|  | Validity of physical activity measure not reported = 0 |
| Was the reliability of the measure assessing physical activity reported | Reliability of physical activity measure reported (or can be acquired externally)  = 1 |
|  | Reliability of physical activity measure not reported = 0 |
| Was the validity of the measure assessing cognitive decline/dementia reported and was it reported to be adequate | Validity of cognitive decline/dementia measure reported (or can be acquired externally) = 1 |
|  | Reliability of cognitive decline/dementia measure not reported = 0 |
| Was the reliability of the measure assessing cognitive decline/dementia reported | Validity of cognitive decline/dementia measure reported (or can be acquired externally) = 1 |
|  | Reliability of cognitive decline/dementia measure not reported = 0 |
| Does the sample consist of ≥1000 participants | The sample consists of ≥ 1000 participants = 1 |
|  | The sample consists of <1000 participants = 0 |
| Are confounders accounted for in the analyses (where appropriate) | Confounders (**age, sex, education**) accounted for in the analyses = 1 |
|  | Confounders (**age, sex, education**) not accounted for in the analyses = 0 |
