# Supplementary material for: Does physical activity prevent cognitive decline and dementia?: A systematic review and meta-analysis of longitudinal studies
Source: BMC Public Health. 2014 May 27;14:510. doi: 10.1186/1471-2458-14-510 (PMC4064273; doi:10.1186/1471-2458-14-510)
Supplement: Additional file 3: Table S3 — Study methodological quality. [file 1471-2458-14-510-S3.docx]

**Table S3** Study methodological quality

| Author | Participation rate | Follow -up duration | Final dropout rate | Dropout profile | Valid PA measure | Reliable PA measure | Valid CD/D measure | Reliable CD/D measure | No. of participants | Confounders | Total |
| --- | --- | --- | --- | --- | --- | --- | --- | --- | --- | --- | --- |
| Ho [[22](#_ENREF_22)] | 0 | 0 | 1 | 1 | 1 | 0 | 1 | 1 | 0 | 1 | 6/10  60% |
| Ho [[22](#_ENREF_22)] | 0 | 0 | 1 | 1 | 1 | 0 | 1 | 1 | 0 | 1 | 6/10  60% |
| Laurin [[23](#_ENREF_23)] | 0 | 1 | 1 | 0 | 1 | 1 | 1 | 1 | 1 | 1 | 8/10  80% |
| Laurin [[23](#_ENREF_23)] | 0 | 1 | 1 | 0 | 1 | 1 | 1 | 1 | 1 | 1 | 8/10  80% |
| Schuit [[24](#_ENREF_24)] | 1 | 0 | 1 | 0 | 1 | 1 | 1 | 1 | 0 | 1 | 7/10  70% |
| Yaffe [[25](#_ENREF_25)] | 0 | 1 | 1 | 0 | 1 | 1 | 1 | 1 | 1 | 1 | 8/10  80% |
| Pignatti [[26](#_ENREF_26)] | 0 | 1 | 0 | 0 | 0 | 0 | 1 | 1 | 0 | 0 | 3/10  30% |
| Lytle [[27](#_ENREF_27)] | 0 | 1 | 1 | 0 | 0 | 0 | 1 | 1 | 1 | 1 | 6/10  60% |
| Flicker [[28](#_ENREF_28)] | 1 | 1 | 0 | 0 | 0 | 0 | 1 | 1 | 1 | 1 | 6/10  60% |
| Singh-Manoux [[29](#_ENREF_29)] | 1 | 1 | 1 | 0 | 0 | 0 | 0 | 0 | 1 | 1 | 5/10  50% |
| Sumic [[30](#_ENREF_30)] | 0 | 1 | 1 | 0 | 1 | 0 | 1 | 1 | 0 | 1 | 6/10  60% |
| Sumic [[30](#_ENREF_30)] | 0 | 1 | 1 | 0 | 1 | 0 | 1 | 1 | 0 | 1 | 6/10  60% |
| Middleton [[31](#_ENREF_31)] | 0 | 1 | 1 | 0 | 1 | 1 | 1 | 1 | 1 | 1 | 8/10  80% |
| Niti [[32](#_ENREF_32)] | 1 | 0 | 0 | 0 | 0 | 0 | 1 | 1 | 1 | 1 | 5/10  50% |
| Etgen [[33](#_ENREF_33)] | 0 | 0 | 1 | 0 | 0 | 0 | 1 | 1 | 1 | 0 | 4/10  40% |
| Iwasa [[47](#_ENREF_47)] | 0 | 1 | 1 | 0 | 0 | 0 | 1 | 1 | 0 | 1 | 4/10 (40%) |
| Lee [[48](#_ENREF_48)] | 0 | 1 | 0 | 0 | 1 | 1 | 1 | 1 | 0 | 1 | 6/10  60% |
| Middleton [[49](#_ENREF_49)] | 0 | 1 | 1 | 0 | 1 | 1 | 1 | 1 | 0 | 1 | 7/10  70% |
| Morgan  [[50](#_ENREF_50)] | 1 | 1 | 1 | 0 | 1 | 1 | 1 | 1 | 1 | 0 | 8/10  80% |
| Verghese [[51](#_ENREF_51)] | 0 | 1 | 1 | 0 | 0 | 1 | 0 | 0 | 0 | 0 | 3/10  30% |
| Yoshitake [[35](#_ENREF_35)] | 1 | 1 | 1 | 0 | 0 | 0 | 1 | 1 | 0 | 0 | 5/10  50% |
| Fabrigoule  [[36](#_ENREF_36)] | 0 | 0 | 1 | 0 | 0 | 0 | 1 | 1 | 1 | 0 | 4/10  40% |
| Laurin  [[23](#_ENREF_23)] | 0 | 1 | 1 | 0 | 1 | 1 | 1 | 1 | 1 | 1 | 8/10  80% |
| Laurin  [[23](#_ENREF_23)] | 0 | 1 | 1 | 0 | 1 | 1 | 1 | 1 | 1 | 1 | 8/10  80% |
| Wilson  [[37](#_ENREF_37)] | 1 | 0 | 1 | 1 | 0 | 0 | 1 | 1 | 0 | 1 | 6/10  60% |
| Verghese  [[38](#_ENREF_38)] | 0 | 1 | 1 | 0 | 0 | 1 | 1 | 1 | 0 | 1 | 6/10  60% |
| Abbott [[39](#_ENREF_39)] | 1 | 1 | 1 | 0 | 0 | 0 | 1 | 1 | 1 | 1 | 7/10  70% |
| Podewils [[40](#_ENREF_40)] | 0 | 1 | 1 | 0 | 1 | 1 | 1 | 1 | 1 | 1 | 8/10  80% |
| Rovio [[41](#_ENREF_41)] | 0 | 1 | 1 | 0 | 0 | 0 | 1 | 1 | 1 | 1 | 6/10  60% |
| Larson [[42](#_ENREF_42)] | 0 | 1 | 1 | 1 | 0 | 0 | 1 | 1 | 1 | 0 | 6/10  60% |
| Bowen [[21](#_ENREF_21)] | 0 | 1 | 1 | 0 | 1 | 0 | 1 | 0 | 0 | 1 | 5/10  50% |
| Buchman [[52](#_ENREF_52)] | 0 | 0 | 1 | 0 | 1 | 1 | 1 | 1 | 0 | 1 | 6/10  60% |
| Chang [[53](#_ENREF_53)] | 1 | 1 | 1 | 0 | 0 | 0 | 1 | 1 | 1 | 1 | 7/10  70% |
| De Bruijn [[54](#_ENREF_54)] | 1 | 1 | 1 | 0 | 1 | 1 | 1 | 1 | 1 | 1 | 9/10  90% |
| Gureje [[55](#_ENREF_55)] | 1 | 0 | 0 | 1 | 1 | 1 | 1 | 1 | 1 | 1 | 8/10  80% |
| Kim [[56](#_ENREF_56)] | 0 | 0 | 1 | 1 | 0 | 0 | 1 | 1 | 0 | 1 | 5/10  50% |
| McCallum [[57](#_ENREF_57)] | 1 | 1 | 0 | 0 | 0 | 0 | 1 | 1 | 1 | 0 | 5/10  50% |
| Morgan [[50](#_ENREF_50)] | 1 | 1 | 1 | 0 | 1 | 1 | 1 | 1 | 1 | 0 | 8/10  80% |
| Ravaglia [[58](#_ENREF_58)] | 1 | 0 | 1 | 0 | 0 | 0 | 1 | 1 | 0 | 1 | 5/10  50% |
| Rovio [[59](#_ENREF_59)] | 1 | 1 | 1 | 0 | 0 | 0 | 1 | 1 | 1 | 1 | 7/10  70% |
| Scarmeas [[60](#_ENREF_60)] | 0 | 1 | 1 | 1 | 1 | 1 | 1 | 1 | 1 | 1 | 9/10  90% |
| Taaffe [61] | 1 | 1 | 1 | 0 | 0 | 0 | 1 | 1 | 1 | 1 | 7/10  70% |
