# Supplementary material for: Does physical activity prevent cognitive decline and dementia?: A systematic review and meta-analysis of longitudinal studies
Source: BMC Public Health. 2014 May 27;14:510. doi: 10.1186/1471-2458-14-510 (PMC4064273; doi:10.1186/1471-2458-14-510)
Supplement: Additional file 4: Table S4 — Sensitivity analyses. [file 1471-2458-14-510-S4.docx]

**Table S4** *Sensitivity analyses*

|  | Cohorts, *n* | High level of physical activity, RR (95% CI) |
| --- | --- | --- |
| Cognitive decline |  |  |
| All | 21 | 0.65 (0.55-0.76) |
| *Study quality* |  |  |
| Low | 13 | 0.56 (0.45 – 0.69) |
| High | 8 | 0.73 (0.59 – 0.91) |
| *Effect size type* |  |  |
| OR | 16 | 0.67 (0.57 – 0.78) |
| RR* | 5 | 0.47 (0.27-0.83) |
| *Adjustments* |  |  |
| <10 | 12 | 0.61 (0.50 - 0.74) |
| ≥10 | 9 | 0.68 (0.51 – 0.91) |
| *Follow-up time* |  |  |
| <10 years | 16 | 0.60 (0.52 – 0.70) |
| ≥ 10 years | 5 | 0.62 (0.52 – 1.27) |
| Dementia |  |  |
| All | 26 | 0.86 (0.76-0.97) |
| *Study quality* |  |  |
| *Low* | 11 | 0.80 (0.62 – 1.04) |
| *High* | 15 | 0.87 (0.79 – 0.96) |
| *Effect size type* |  |  |
| OR | 12 | 0.89 (0.72 – 1.10) |
| RR | 14 | 0.84 (0.74 – 0.95) |
| *Adjustments* |  |  |
| <10 | 13 | 0.83 (0.66 – 1.05) |
| ≥10 | 13 | 0.86 (0.77 – 0.96) |
| *Follow-up time* |  |  |
| <10 years | 19 | 0.85 (0.73 – 0.99) |
| ≥ 10 years | 7 | 0.86 (0.68 – 1.11) |
| *Excluding Wilson et al.* | 25 | 0.82 (0.73-0.91) |

*Note RR category includes all effect sizes other than ORs; OR: Odds Ratio; RR: Relative Risk
